# Anaplastic lymphoma kinase aberrations correlate with metastatic features in pediatric rhabdomyosarcoma

**DOI:** 10.18632/oncotarget.10368

**Published:** 2016-07-01

**Authors:** Patrizia Gasparini, Michela Casanova, Raffaella Villa, Paola Collini, Rita Alaggio, Angelica Zin, Paolo Bonvini, Cristina R Antonescu, Renata Boldrini, Roberto Caserini, Massimo Moro, Giovanni Centonze, Cristina Meazza, Maura Massimino, Luca Bergamaschi, Roberto Luksch, Stefano Chiaravalli, Gianni Bisogno, Nadia Zaffaroni, MariaGrazia Daidone, Gabriella Sozzi, Andrea Ferrari

**Affiliations:** ^1^ Tumor Genomics Unit, Fondazione IRCCS Istituto Nazionale dei Tumori, Milan, Italy; ^2^ Department of Pediatric Oncology, Fondazione IRCCS Istituto Nazionale dei Tumori, Milan, Italy; ^3^ Biomarkers Unit, Fondazione IRCCS Istituto Nazionale dei Tumori, Milan, Italy; ^4^ Soft Tissue and Bone Pathology, Histopathology and Pediatric Pathology Unit, Fondazione IRCCS Istituto Nazionale dei Tumori, Milan, Italy; ^5^ Department of Pathology, University Hospital of Padova, Padova, Italy; ^6^ Institute of Pediatric Research Città della Speranza, Padova, Italy; ^7^ Molecular Pharmacology Unit, Fondazione IRCCS Istituto Nazionale dei Tumori, Milan, Italy; ^8^ Department of Pathology, Memorial Sloan Kettering Cancer Center, New York, NY, USA; ^9^ Department of Pathology, Bambino Gesù Children Hospital-Research Institute, Rome, Italy; ^10^ Hematology Oncology Division, Department of Mother and Child's Health, University Hospital of Padova, Padova, Italy

**Keywords:** anaplastic lymphoma kinase, rhabdomyosarcoma, EML4-ALK, chromosomal rearrangement, metastasis

## Abstract

Rhabdomyosarcoma (RMS) is the most frequent soft tissue tumor in childhood and arises from immature mesenchymal cells committed to skeletal muscle differentiation. Anaplastic Lymphoma Kinase (ALK) is a receptor tyrosine kinase aberrantly expressed in several cancers. Moreover, ALK full-length receptor protein has been observed in RMS, although its clinical and functional significance is yet controversial. The role of ALK and its clinical relevance were investigated in a selected cohort of 74 FFPE pediatric RMS and a panel of RMS cell lines, evaluating its gene and protein status, utilizing Fluorescent *In Situ* Hybridization (FISH), immunohistochemistry (IHC) and Western blot approaches. Moreover, to get insight into its possible therapeutic relevance, effects of ALK silencing on cell proliferation, invasion and apoptosis were studied in RMS cells. ALK IHC positivity was significantly correlated with gene copy number gain, the alveolar subtype, PAX3/7-FOXO1 rearrangements, the presence of metastasis at diagnosis and a worse overall outcome. Furthermore, EML4-ALK fusion gene associated with higher protein expression was identified in an embryonal RMS. ALK silencing in RH30 ALK positive cells strongly inhibited invasion capability. Overall, our data suggest a potential role of ALK in pediatric RMS.

## INTRODUCTION

Rhabdomyosarcoma (RMS) is one of the typical embryonal tumor of childhood, representing about 50% of soft tissue sarcomas of pediatric age. RMS is a high grade malignant disease with a marked propensity to metastasize. However, these tumors generally are responsive to the modern multimodal therapeutic approaches including multidrug intensive chemotherapy, to the point that three out of four patients with localized RMS may be currently cured [1, 2, 3]. Yet, the prognosis of RMS depends on multiple variables, and for some patients the outcome remains dismal [4]: i.e. patients with alveolar histology continue to have less than optimal outcome, and most patients with distant metastasis or relapsing disease do not achieve long term cure [5]. For these patients’ categories, novel effective therapeutic approaches are absolutely needed [6].

RMS is a sarcoma showing skeletal muscle differentiation (WHO STS 2013). The two main histological subtypes are characterized by different molecular profiles [7, 8]: the embryonal RMS (ERMS) displays a loss of heterozygosity at 11p15 [9], along with the acquisition of chromosome 8 and several other mutations [7, 8], whereas the alveolar RMS (ARMS) is characterized in the vast majority by the t(2;13) translocation and its PAX3-FOXO1 fusion gene, or the less frequent variant t(1;13) and the PAX7-FOXO1 gene [10]. Other rare variants have been described, and approximately 10% of ARMS cases lack evidence of a gene fusion [9, 11, 12].

More recently, various proteins and pathways have been considered in RMS tumorigenesis to identify potentially molecular targets for novel therapy. A putative target for various malignancies is the anaplastic lymphoma kinase (ALK) gene. ALK has been identified in anaplastic large cell lymphoma as chromosomal translocation t(2;5)(p23;q35) involving the entire nucleophosmin (NPM) gene on chromosome 5 and the 3′ portion of the ALK gene on chromosome 2, which generates the oncogenic NPM-ALK kinase. Nevertheless, ALK can be both detected as a fusion protein kinase or full length receptor protein in several tumour types [13, 14, 15] where it activates a multitude of downstream signaling pathways following its own activation and phosphorylation.

The expression of full-length ALK receptor has been observed also in RMS [16], however, its involvement in tumor onset and progression has not yet been comprehensively investigated. Data from the literature suggests ALK being expressed mainly in ARMS, and in metastatic ERMS with poor prognostic implications [17, 10, 18]. Moreover, literature reports a correlation between the ALK protein expression with a genetic copy number acquisition of ALK [10, 16, 19, 20, 21, 22]. Interestingly, only a few studies reported true amplification [21, 22] and rearrangements of the ALK gene [19], in isolated RMS cases.

We investigated the clinicopathologic correlations and prognostic role of ALK genomic status and protein expression in a series of 74 pediatric RMS, with known FOXO1 translocation status, by immunohistochemistry (IHC) and fluorescence *in situ* hybridization (FISH). Moreover, to get insight into its possible therapeutic relevance, the effects of ALK silencing on cell proliferation, invasion and apoptosis were studied in a panel of RMS cell lines with distinct patterns of ALK status.

## RESULTS

### Characterization of ALK in RMS cell lines

A panel of RMS cell lines was analyzed for *ALK* gene copy number by FISH and ALK protein expression by IHC or Western Blotting (WB) in an attempt to identify experimental models characterized by distinct patterns of ALK status (Supplementary Table 1). However, RMS cell lines neither showed *ALK* gene amplification nor protein expression, with the exception of PAX3-FOXO1-positive RH30 cells characterized by a significant acquisition of extra copies of *ALK* gene (5–12 signals), measured by FISH, coupled to a marked protein expression, assessed by both IHC (data not shown) and Western blotting (Supplementary Figure 1). Nevertheless, despite its expression, no active/phosphorylated form of mature (200 kDa), immature (120 kDa) or rearranged ALK protein (80 kDa) was detected in these cells (Supplementary Figure 1).

### Characterization of ALK in RMS patients’ cohort: clinical and pathological features at diagnosis

In order to study the role of ALK in RMS, a total of 74 RMS specimens were selected, characterized with respect to ALK gene and protein status and correlated with their clinic-pathological information available (Table 1).

**Table 1:** Correlation of ALK protein and gene status with clinic-pathological features

		IHC			FISH		
Histology	Total (%)	Positive (%)	Negative (%)	*p* value	Positive (%)	Negative (%)	*p* value
Alveolar	28 (38)	22 (79)	6 (21)	0.0001^a^	18 (64)	10 (36)	0.054
Embrional	46 (62)	11 (20)	35 (76)		18 (39)	28 (61)	
**Translocation Status**							
RMS-t^b^	16 (22)	15 (94)	1 (6)	0.0001	14 (90)	2 (10)	0.001
RMS-non	58 (78)	18 (31)	40 (69)		23 (40)	35 (60)	
**Age**							
0–10	36 (49)	13(36)	23 (64)	0.17	17 (47)	19 (53)	0.8
11–24	38 (51)	20(53)	18 (47)		20 (53)	18 (47)	
**Sex**							
Female	29 (39)	14 (48)	15 (52)	0.639	15 (52)	14 (48)	1
Male	45 (61)	19 (43)	26 (57)		22 (51)	23 (49)	
**Stage (IRS)**							
1–2	21 (28)	7 (33)	14 (67)	0.301	8 (38)	13 (62)	0.302
3–4	53 (72)	26 (49)	27 (51)		29 (54)	24 (46)	
**Tumor Invasiveness**							
T1	32 (43)	15 (47)	17 (53)	0.8	18 (56)	14 (44)	0.481
T2	42 (57)	18 (43)	24 (57)		19 (45)	23 (55)	
Lymph Node Involvement							
N0	57 (77)	22 (39)	35 (61)	0.094	28 (49)	29 (51)	1
N1	17 (23)	11 (65)	6 (35)		9 (53)	8 (47)	
Metastatic Disease							
M0	56 (76)	19 (34)	37 (66)	0.002	26 (46)	30 (54)	0.592
M1	18 (24)	14 (78)	4 (22)		10 (56)	8 (44)	
**OS^c^**							
Dead	22 (30)	14 (65)	8 (35)	0.0387	13 (59)	9 (41)	0.446
Alive	52 (70)	18 (35)	34 (65)		24 (46)	28 (54)	

a *p* values in bolt are statistically significant.

b Translocated rhabdomyosarcomas (PAX3/7-FOXO1).

c Overall Survival.

### Protein and genomic status of ALK

**Figure 1:**
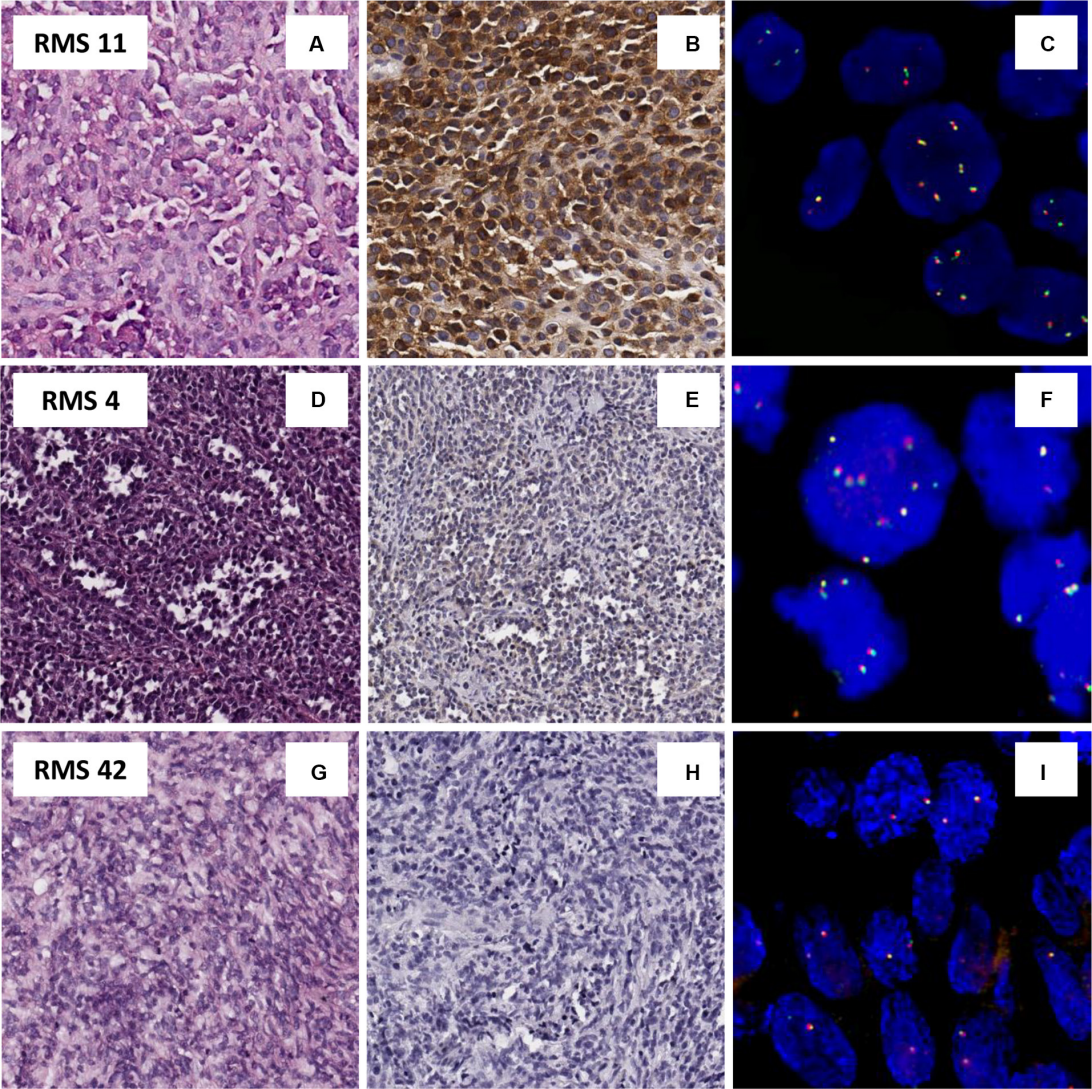
Protein an genetic status of ALK (**A**–**C**) Example of pediatric alveolar rhabdomyosarcoma, RMS 11(A), positive for ALK protein by IHC (B) associated with an acquisition of gene copy number of ALK by FISH (C). (**D**–**F**) Example of alveolar rhabdomyosarcoma, RMS 4, (D) with a ALK focal positivity IHC (E), and an acquisition of ALK copy number only in IHC-ALK positive tumor cells (F). (**G**–**I**) Example of embryonal rhabdomyosarcoma, RMS 42 (G), ALK IHC negative (H) and disomic with no gene copy number gain by FISH (I).

With respect to protein expression, an overall ALK positivity was identified in 33/74 (45%) RMS cases. In details, cytoplasmic reactivity was present in 22/28 (79%) ARMS (Figure 1A–1B), 15/16 (93%) of which with PAX3/7-FOXO1 fusion gene, and in 11/46 (24%) ERMS. Moreover, ALK immunoreactivity was strong and diffuse in 24/33 (73%) cases, and focal in 9/33 (27%) cases (5 ARMS and 4 ERMS) (Figure 1D–1E). Forty-one cases (55%) were totally negative to ALK staining (Figure 1G–1H).

Likewise, FISH analysis of the whole series revealed no true amplification of the ALK gene but a recurrent copy number gain that matched perfectly with the previously assessed protein status. Among the 74 RMS analyzed, 36/74 (49%) revealed a gene copy number gain (> 6). In contrast, 18/28 (64%) ARMS showed an average gain varying from 2.9 to 4.8 and a percentage of tumor cells with extra copies of *ALK* ranging from 51% to 100% (Figure 1A, 1C), while 18/46 (40%) ERMS showed an average of signals per cells varying from 2.8 to 4.4, with a percentage of positive cells ranging from 43% to 100%. The remaining 10 ARMS and 28 ERMS were described as disomic for the ALK gene, with an average of FISH signals of 2-2.4 (percentage of tumor cells with acquisition 2–11%) and 2–2.7 (percentage of tumor cells with acquisition 1–57%), respectively (Figure 1 panel 1G and 1I). Besides, all RMS cases characterized by IHC as focal ALK immunoreactivity (5 ARMS, and 4 ERMS) resulted with an ALK copy number gain restricted to IHC-positive areas (Figure 1 panels 1D and 1F). FISH analysis of the chromosome 2 centromere in all cases classified by FISH as ‘ALK gene copy number gain’ showed that ALK gene copy number gain in RMS tumor cells was associated with polysomy of chromosome 2.

### EML4-ALK rearranged ERMS

**Figure 2:**
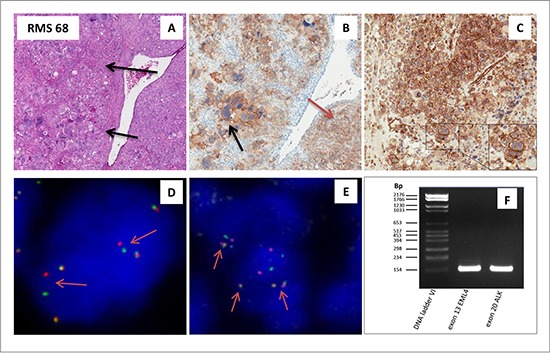
ALK rearranged ERMS (**A**–**F**) Embryonal rhabdomyosarcoma (RMS 68) with an anaplastic component (arrow) (A)characterized by an EML4-ALK rearrangement. Positivity for ALK protein by IHC is present in the membrane within the anaplastic component as indicated by the black arrow, and within the cytoplams as indicated by the red arrow (B). Positivity for phospho-ALK by IHC is observed in the membrane as well as the cytoplasm (C). FISH analysis utilizing a split apart commercial probe, revealed the presence of an ALK rearrangement as represented by the single red and green signals (as indicated by the arrows; D). EML4-ALK rearrangement was confirmed by FISH, utilizing a EML4-ALK fusion commercial probe. The presence of the fusion yellowish signal (arrows) indicate the fusion gene between the EML4 and ALK gene (E). EML4-ALK fusion transcript as visualized with rt-PCR: lane 1 DNA ladder VI, lane 2 exon 13 of EML4 breakpoint at 155bp and lane 3 exon 20 of ALK breakpoint at 155 bp (F).

IHC analysis, however, revealed one single ERMS case, in which a strong ALK signal decorated the membrane of some morphologically distinguishable anaplastic cells, whereas the more typical cytoplasmic staining was confined to the remaining neoplastic component (Figure 2A–2B). Surprisingly, FISH analysis revealed an ALK structural rearrangement coupled to copy number gain in the 100% of the analyzed tumor cells (Figure 2D), suggesting the existence of an ALK truncated variant in this patient. To identify the partner gene fused to *ALK*, RT-PCR analysis was performed on the FFPE material available, using primers specific for the most common *ALK* fusion variants so far detected. The analysis demonstrated the presence of the EML4-ALK inv(2) (p21p23) (exon 13 of EML4 and exon 20 of ALK breakpoints at 155 bp) (Figure 2F), which was further confirmed by Sanger sequencing (data not shown) and FISH (Figure 2E). Additionally, *p*-ALK IHC resulted positive, with a similar staining pattern of ALK, confirming the presence of the activated form of ALK in this rearranged case (Figure 2C).

### Clinical correlations and prognostic implications

**Figure 3:**
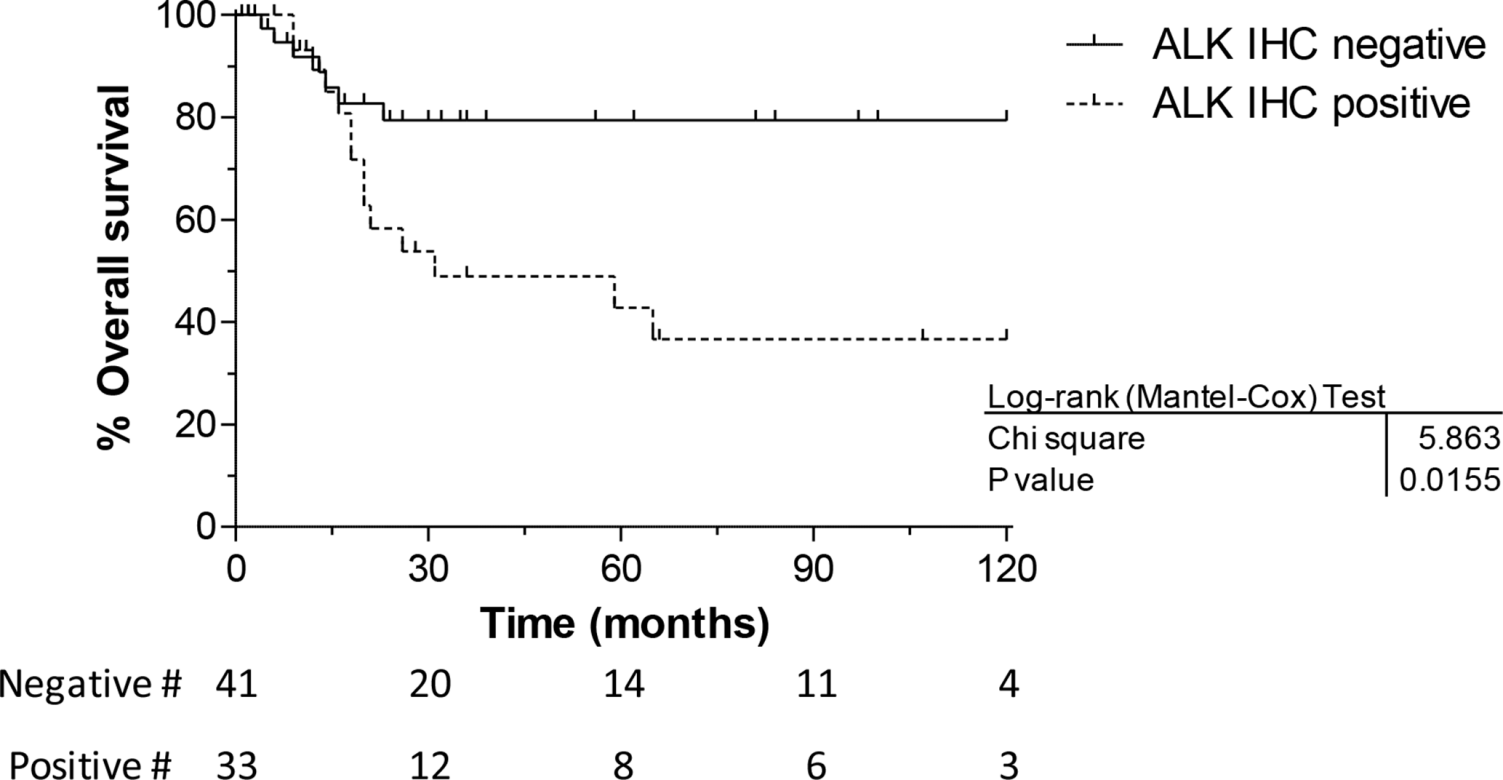
Overall survival vs immunohistochemistry The Kaplan Meier graph clearly shows that RMS with ALK IHC positivity have a worse overall outcome compared to those cases with no protein expression.

Both IHC and FISH methodologies resulted effective to identify aberrations in the ALK protein and gene status, and Fishers’ exact test confirmed the significant association between the two techniques (*p* = 0.0001). Moreover, a K coefficient of 0.73, a quantitative measurement of the agreement between the two assays, confirmed the good association between IHC and FISH analysis (Table 2), as demonstrated when ALK expression and gene copy number variation were measured by IHC and FISH, respectively, in RMS specimens distinguished by histology (11/46 ERMS and 18/28 ARMS; *p* = 0.0001), fusion gene status (RMS t vs RMS non; *p* = 0.0001), presence of metastasis (*p* = 0.002) and overall survival (*p* = 0.039) (Table 1 and Figure 3).

**Table 2:** Correlation between IHC and FISH methodologies

		FISH		
		Positive (> 2.8)^a^	Negative (< 2.8)^b^	Total
**IHC**	Positive	30	3	33
	Negative	8	33	41
	**Total**	38	36	74

Fishers’ test: *p* = 0.0001.

K coefficient = 0.73.

a ‘positive’ for copy number gain when average of signals per cells was of > 2.8.

b ‘negative’ for copy number gain when average of signals per cells was of < 2.8.

Besides, patient outcome analysis showed that histology, age and disease stage were significantly associated with survival in univariate analysis (Table 3), likewise ALK expression, when outcome of ALK positive and negative patients were compared (HR 2.86, 95% CI 1.20–6.85, *p* = 0.018) (Table 3A and Figure 3).

**Table 3A:** Univariate analysis of patient outcome

	#cases	%	HR (95% CI)	*p* Value
**AGE**				
< 10 yrs	35	**72**	3.74 (1.35–10.37)	**.0113**
≥ 10 yrs	39	**39**		
**IRS**				
I–II	19	**100**		**<.0001**
III	35	**53**		
IV	19	**0**		
**HISTOLOGY**				
ERMS	46	**79**	3.84 (1.58–9.36)	**.003**
ARMS	28	**17**		
**ALK-IHC**				
negative	40	**68**	2.86 (1.20–6.85)	**.018**
positive	34	**35**		

Bivariate analysis further confirmed ALK expression as an unfavourable factor for young adolescents (HR 3.25 for present vs absent ALK expression, 95% CI 1.07–9.90, *p* = 0.038) as, though to a lesser extent, for patients with stage III tumors at diagnosis (HR 3.34 for positive vs absent ALK expression, 95% CI 0.83–13.46, *p* = 0.09) (Table 3B). At last, ALK IHC positive cases have a worse overall survival regardless their fusion status. ALK positive RMS-non have a prognosis just as poor as the RMS-t. Moreover, only one RMS-non is ALK IHC negative and this case has a explicit worse OS compared to all the other ALK negative with no fusion positive. In conclusion, ALK positivity is independent from the fusion status.

**Table 3B:** Bivariate analysis of patient outcome and ALK protein status

	ALK-negative		ALK-positive			
Variables	#cases	%	#cases	%	HR (95% CI)	*p* Value
**AGE**						
< 10 yrs	22	72	13	83	0.73 (0.08–7.06)	.79
≥ 10 yrs	18	74	21	20	3.25 (1.07–9.90)	.038
**IRS**						
I–II	12	100	7	100		
III	22	69	13	33	3.34 (0.83–13.46)	.09
IV	5	0	14	0	0.53 (0.15–1.83)	.31
**HISTOLOGY**						
ERMS	34	82	12	67	1.49 (0.29–7.76)	.63
ARMS	6	33	22	24	2.36 (0.53–10.50)	.26
**FUSION STATUS**						
RMS-t	1	1	15	35	0.08 (0.005–1.229)	.069
RMS-non	39	70	19	35	2.70 (0.94–7.78)	.066

### Effects of siRNA-mediated ALK silencing in the RH30 cell line

**Figure 4:**
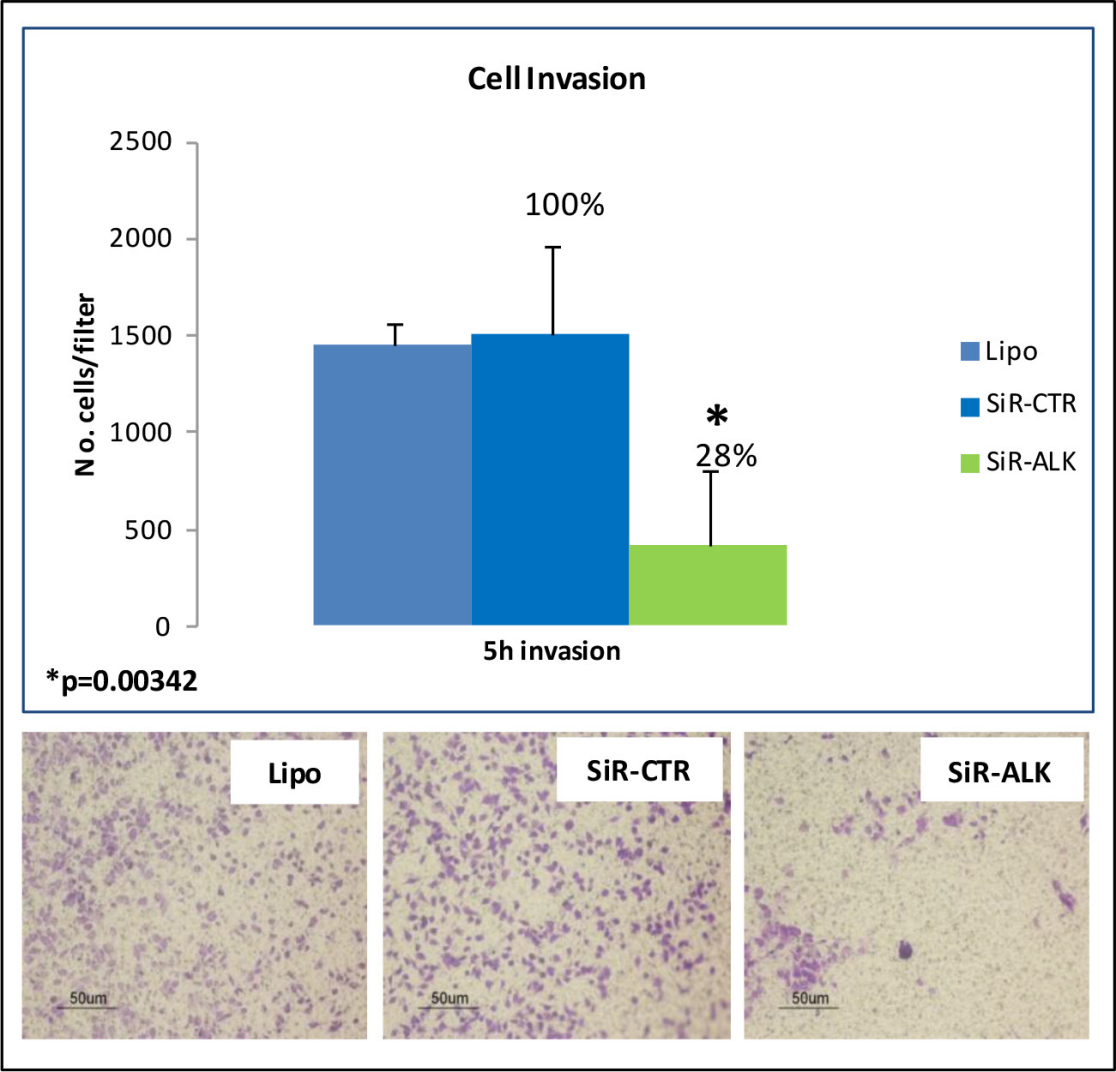
ALK silencing: siRNA-mediated down-modulation of ALK significantly inhibited invasion capability of RH30 cells

Finally, to investigate the functional role of ALK in RMS cells, a siRNA-binding a specific consensus sequence within the open reading frame of ALK mRNA (siR-ALK), and a control siRNA (siR-CTR) were used. The results reported in Supplementary Figure 2 demonstrate that transfection of RH30 cells wih siR-ALK resulted in an appreciable reduction of ALK expression at both mRNA and protein level. In addition, siRNA-mediated ALK silencing markedly inhibited tumor cell invasion (72% of reduction compared to siR-CTR) (Figure 4). Conversely, ALK down-modulation did not induce changes in cell proliferation and morphology (Supplementary Figure 2) as well as in the number of apoptotic cells (data not shown). Our data suggest a potential role of ALK in RMS cell spreading rather than in proliferation or survival (Figure 4).

## DISCUSSION

The developing of novel therapies for RMS is a critical clinical need. To date, the status of ALK in RMS has been studied by several groups [16, 21, 22], since its protein expression in clinical samples might support the use of ALK small molecule inhibitors as an appealing therapeutic option. However, the relevance of ALK as a druggable target in this malignancy is still unclear and warrants further investigation. Our study, reveal a strong association of ALK protein expression with a gene copy number gain, more frequently observed in ARMS than in ERMS. Moreover, ALK IHC positivity was significantly correlated with the alveolar subtype, PAX3/7-FOXO1 rearrangements, the presence of metastasis at diagnosis and a worse overall outcome.

The significance of ALK expression in RMS remains debatable. Indeed, expression of ALK in RMS, assessed by IHC, varied consistently among previously reported studies, ranging from 53% [21] to 81% [10] in ARMS, and from 6% [22] to 32% [10] in ERMS. Similarly, the *ALK* gene status measured by FISH/CISH was reportedly somehow different; whilst Yoshida et al. observed uncommon (18%) ALK gene copy number variations, others reported much more frequent event (ARMS 45%; ERMS 61%) [10].

In our series, an overall 43% of specimen presented an ALK protein positivity (79% of ARMS and 26% of ERMS) with a statistically strong association with a gene copy number gain of ALK. Moreover, a careful IHC analysis of these cases identified a subgroup of samples with an ALK focal positivity, a faint staining decoration in small delimited areas, as opposed to the majority of the cases displaying a strong positive cytoplasmatic staining throughout the sample. Interestingly, an ALK focal positivity was associated with an ALK gene copy number gain exclusively in the positive regions.

Similarly, clinical implications of ALK expression in RMS are also not uniform. In some studies, ALK expression correlates with tumour histology and metastases at presentation, but not with overall survival [22]; others, instead, showed a prognostic significance only in ERMS displaying ALK gene copy number gain [10]. In our study, the correlation of ALK expression with patients’ clinic-pathological variables was confirmed, while a significant association with alveolar subtype, presence of metastases and worse outcome, was observed. Noteworthy, the prognostic relevance of ALK expression was also seen in specific subsets, as adolescents or unresected cases. Lastly, ALK positivity is independent from the fusion status: ALK positive RMS-non translocated have a prognosis just as poor as the RMS-translocated.

Whereas most of the studies report a correlation between ALK protein expression and a copy number variation of ALK [10, 16, 19, 20, 21, 22] a few identified a true amplification in isolated patients [21, 22], but only one study described ALK rearrangements in three RMS [19].

Herein, we describe for the first time a rearrangement of ALK, EML4-ALK inv(2) (p21p23) so far described in other tumor types [23], but not in RMS. ALK-positivity by IHC of this ERMS was observed as a strong positive membrane decoration in the anaplastic component and a more common cytoplasmic staining in all the remaining tumor cells. Although IHC seems to be a valid method to screen ALK expression in this malignancy, these findings imply the need of a careful and combined analysis of both the genetic and protein status of ALK, as a gene rearrangements may have different consequences to tumour cell growth and survival compared to copy number gain. We further demonstrate an ALK phosphorylation by IHC of this rearranged case, indicative of a possible response to specific small molecule inhibitors, as reported in other tumor types [24].

So far, the functional role of ALK in RMS still remains unknown. Our study along with others [17, 10, 25], confirms that ALK receptor activity may be hard to be detected *in vitro*. Only few studies reported that the phosphorylation of ALK receptor in ALK-positive RMS cells was feasible only after stimulation with agonist antibodies [17, 25]. Accordingly, we were unable to show basal ALK phosphorylation in unstimulated RMS cell lines, although our *in vitro* analyses demonstrated the consequences of ALK silencing on RMS cells invasion capacity. ALK overexpression, hence, may have evident biological effects on RMS cells invasion ability, even in its unphosphorylated form.

Overall, our data suggest a probable role of ALK in tumor progression and metastasis of pediatric RMS. In consideration of its function played in other tumor types such as lung cancer and neuroblastoma, the possible therapeutic role of ALK in RMS needs to be further investigated in *in vitro* models and, ultimately, in *in vivo* pharmacological studies.

## MATERIALS AND METHODS

### Cell lines

A panel of 12 RMS cell lines has been analyzed for ALK gene by FISH and, for ALK protein expression, by IHC and/or Western Blotting (WB). In details, RH30 and RH4 (purchased from ATCC), were ARMS with PAX3-FOXO1 fusion transcript, CW9019 (purchased from ATCC), was ARMS with PAX7-FOXO1 fusion transcript; TE381, RD, TE441, RH36, RH18, TE671, TE441, RD-M1 xenograft, RD-M2 xenograft (purchased from ATCC) were ERMS; A204 (purchased from ATCC) was a rhabdoid tumor. Moreover, a lung adenocarcinoma cell line H2228 (purchased from ATCC) was utilized as a positive control for ALK rearrangement (EML4-ALK).

### Pediatric RMS case series

A total of 74 pediatric RMS (28 ARMS and 46 ERMS) from untreated patients aged up to 24 years, with known clinical data at onset and follow-up until July 2014 were retrieved and studied for FISH and IHC analysis. Fifty two cases were recovered from the files of the Department of Diagnostic Pathology and Laboratory Medicine of the Fondazione IRCCS Istituto Nazionale dei Tumori of Milan, and 23 RMS from the Pathology Department of the University Hospital of Padova. The study was approved by the Internal Review Board and the Ethics Committee of both the institutions. All patients gave their written consent for diagnosis and research activities when they were admitted to the hospital.

All cases were already investigated for the presence of PAX3/7-FOXO1 translocation. In all cases formalin-fixed paraffin-embedded (FFPE) material was available for reclassification, following the updated WHO criteria for soft tissue sarcomas (2013) into ERMS and ARMS by two experts pediatric sarcoma pathologists (PC and RA).

### ALK protein expression by Immunohistochemistry (IHC)

ALK protein expression was investigated by IHC methods both in cell lines and tumor samples. Briefly, three to four micron-thick FFPE sections were unmasked with EDTA buffer at pH8 for 30 minutes, made react with the ALK antibody (clone 5A4, Santa Cruz, Heidelberg, Germany, dilution 1:100 for 60 min) and then incubated with a commercially available detection kit (EnVision™ FLEX+, Dako, Glostrup, Denmark) in an automated immunostainer (Dako Autostainer System). IHC results were scored as positive (more than 10% reactive neoplastic cells) or negative (10% or less reactive neoplastic cells). Positive results were further defined on the basis of the distribution of reactivity (focal or diffuse), intensity (weak, moderate, strong), and cellular site (cell membrane, cytoplasm, nucleus). Positive and negative controls were used as appropriate. Moreover, phospho-ALK expression by IHC was studied in the ALK rearranged case utilizing the antibody Phospho-ALK (tyr 1604, Cell Signaling, dilution 1:5 for 120 min) with a few modification of the protocol (unmasked with Dako PT-link, EnVision™ FLEX Target Retrieval Solution, High pH – 60 mi at 96°C).

### ALK protein expression by Western blotting

Protein expression of all RMS cell lines was also characterized by WB assay, and for those cell lines that resulted IHC ALK positive, its phosphorylated form was also investigated. Samples containing 200 μg of protein per lane were separated on precast 4–12% NuPAGE bis-tris gels (Invitrogen), and were transferred onto Hybond ECL nitrocellulose membranes (Invitrogen) using the NuPAGE transfer buffer and iBlot device (Invitrogen). Nitrocellulose membranes were blocked in PBS-Tween 20 with 5% skim milk, first incubated with the primary antibodies (Cell Signaling) and then with the secondary peroxidase linked whole antibodies (GE Healthcare Europe). Bound antibodies were detected using the Super Signal chemiluminescent substrate (GE Healthcare Europe). β-Actin monoclonal antibody (Abcam) was used to confirm equal protein loading on the gel. Filters were autoradiographed, and autoradiographs were scanned and quantified by densitometric analysis using Vision Works LS software (UVP, Upland, CA, USA). The H2228 lung adenocarcinoma cell line, positive for ALK rearrangement (EML4-ALK), was utilized as positive control for ALK expression (total protein and phosphorylated form at 80kDa).

### Genomic status of ALK by fluorescent *in situ* hybridization

FISH analysis was performed on areas selected by the pathologists as being representative of tumor cells and of the regions positive for protein expression of ALK, of 2–4 μm-thick paraffin sections of all FFPE RMS tissues and of cell blocks obtained by RMS cell lines, by counting at least 100 tumor cells. Briefly, a commercially available break-apart, dual-color gene specific probe at chromosome 2p23 (Abbott Molecular, Vysis^®^ LSI^®^ ALK Dual Color, Break Apart Rearrangement Probe) was used to identify any ALK rearrangement. The commercial probe was utilized according to manufacturer's instructions. For ALK FISH analysis, two or more orange/green fusion signals (yellowish) indicated cells with wild type configuration, whereas one or more yellowish fusion signals along with separate green and orange signals, or isolated red signals, identified cells with the rearranged gene. Taking into consideration heterogeneity of the tumor, an average of signals per cells were reported for each case, along with the percentage of tumor cells presenting a numeric alteration in gene copy number. In addition, in absence of structural aberration of the gene and of gene clusters, indicative of a true amplification, the term ‘gene copy number gain’ was utilized when the average of signals per tumor cells was > 2.8 and the percentage of cell presenting an acquisition of copies was of more than 40%. FISH analysis was described as ‘positive’ for copy number gain when average of signals per cells was of >2.8 and ‘negative’ when average of signals per cells was of < 2.8. For FISH results presenting a gene copy number gain an additional FISH assay was performed, on adjacent sections, for chromosome 2, utilizing CEP2 probe (centromeric alpha-satellite specific for chromosome 2, from Abbott Molecular, Vysis^®^) to discriminate acquisition of copies rather than true amplification of the gene. At last, a commercial probe ALK/EML4 fusion (Kreatech, Netherlands) was utilized to confirm the fusion transcript EML4-ALK inv(2) (p21p23) identified by RT-PCR.

### Fusion partner by reverse transcription (rt)-PCR analysis

Identification of the fusion partner of rearranged ALK was performed by rt-PCR analysis of the following recurrent ALK partners: t(2;5)(p23;q35) NPM-ALK [26], t(2;22)(p23;q11.2) MYH9-ALK [27], t(1;2)(q25;p23) TPM3-ALK [28], t(2;17)(p23;q23) CLTC1-ALK [29], t(2;2)(p23;q13)inv(2)(p23;p15;q31) RANBP2-ALK [30] and EML4-ALK inv(2) (p21p23) [31]. For this latter one, common primer to exon 20 of ALK was used along with primers to exons 6, 13, and 18 of EML4 in order to determine EML4-ALK breakpoints. ALK exon 20 reverse primer: (TGTCTAACTCGGGAGACTATGAAA). EML4 forward primers: exon 6 (CCTTCAACACCCAAATTAATACC), exon 13 (TATGGAGCAAAACTACTGTAGAGC), exon 18 (CACACAGACGGGAATGAACA). Briefly, total RNA from FFPE tissue was extracted by Absolutely RNA FFPE kit (Stratagene, Santa Clara, California) according to the manufacturer's instructions. One and two micrograms of total RNA was reverse-transcribed by using the EuroScript M-MLV Reverse Transcriptase (RNase H-) (Euroclone, Milan, Italy), 0.5 mM of each dNTPs and random examers. PCR amplification was performed by using the BIOTAQ DNA Polymerase (Bioline, London, UK) according to the manufacturer's instructions. PCR reaction mixture contained 1.5 mM MgCl2, 0.2 μM of each primer, 1× PCR Buffer, 0.4 mM of each dNTPs, 0.5 U of Taq polymerase, and 1 ml of the RT product in a final 20 ml reaction volume. Glyceraldehyde-3-phosphate dehydrogenase (GAPDH) expression was concomitantly assessed as a control for presence of amplifiable RNA and for efficiency of reverse transcription. PCR reaction products were electrophoresed through 2% agarose gels, and their sizes were determined by comparative analysis with DNA Marker VI (Roche, Milan, Italy).

### ALK mRNA expression by real time RT-PCR

Total RNA was extracted from RMS cells using Qiagen RNeasy Mini Kit (Qiagen S.r.l., Milano, Italy) and DNase I-digested. The expression levels of ALK mRNA were assessed by real-time RT-PCR (TaqMan^®^ Assay Hs000608289_m1; Applied Biosystems/Life Technologies Italia), according to standard procedures. Amplifications were run on the 7900HT Fast Real-Time PCR System (Applied Biosystems/Life Technologies Italia). Data were analyzed by SDS 2.2.2 software (Applied Biosystems/Life Technologies Italia) using the 2−ΔΔCt method and reported as Log10 [RQ], where Ct represents the threshold cycle and RQ is the relative quantity with respect to a calibrator sample.

### siRNA-mediated ALK silencing in RMS cells

A siRNA binding the open reading frame of ALK mRNA (positions 5136–5154, GeneBank accession no. NM_004304.4) (SiR-ALK) was used [32]. A control siRNA made up of a scrambled sequence with no significant homology to any known human mRNA was included in this study (siR-CTR). SiRNAs were manufactured by the Eurofins MWG Operon (Ebersberg, Germany) as preformed and purified duplexes.

### Transfection procedures and *in vitro* analyses

The day before transfection, RMS cells were seeded at a density of 8 × 10^5^ per 25 cm^2^ flask. A given amount of each ALK and control siRNA was mixed with Lipofectamine2000 (Invitrogen, San Giuliano Milanese, Italy) for 20 min at room temperature. The mixtures were then applied to the cells in a volume of Opti-MEM I (Invitrogen), giving a final concentration of 100 nM. After a 4-h incubation at 37^°^C, cells were washed with PBS and a culture medium supplemented with serum was added. At different intervals after transfection (from 24 to 72 h), cells were collected by trypsinisation and used subsequently in the different assays. Cells exposed to Lipofectamine2000 alone were referred to as mock control throughout this study.

For apoptosis analysis, adherent cells were pooled together with detached cells and then scored for nuclear morphology of apoptosis (chromatin condensation and DNA fragmentation) by labeling with a solution containing 50 mg/ml of propidium iodide, 50 mg/ml of RNase and 0.05% Nonidet P40 in PBS. After staining, the slides were examined under a fluorescence microscope, and the percentage of cells with an apoptotic nuclear morphology was determined by scoring at least 500 cells in each sample. Each experimental sample was run in triplicate.

In the invasion assay, RMS cells were transferred to the upper chamber of 24-well transwell chambers (Costar, Corning, Inc., Corning, NY) in serum-free medium. The transwell membranes were previously coated with 12.5 μg Matrigel per well (BD Biosciences, San Jose, CA) and dried for 1 h. Conditioned medium, obtained by incubating growing cells in a medium without serum for 24 h, was added to the lower chamber as a chemoattractant. After 24 h of incubation at 37°C, cells that invaded the Matrigel and migrated to the lower chamber were fixed in 95% ethanol, stained with a solution of 0.4% sulforhodamine B in 0.1% acetic acid, and counted under an inverted microscope.

### Statistical analysis

The Chi-square or Fisher's exact (FE) testing, when appropriate, were used to assess differences between subsets defined by ALK expression; investigated categories included patient characteristics (gender, age at onset), tumor type (ARMS versus ERMS), presence versus absence of either PAX3-FOXO1 or PAX7-FOXO1 translocation, stage (IRS), TNM classification, state at the last control (dead versus alive). The precision of the agreement between ALK protein expression by IHC and results of FISH analysis was assessed by Cohen's Kappa statistics.

The median follow-up of the case series was 23 months (range 1–250 months), and at the time of the last follow-up date 22 patients were dead. Overall survival (OS) was calculated from date of enrollment to the date of death due to any cause, or censored at the date of last follow-up for living patients. OS was estimated using the Kaplan-Meier method and compared across the groups using the log-rank test. For clinic-pathologic features and ALK expression hazard ratios (HRs) together with 95% confidence intervals (CIs) were estimated by fitting the Cox survival model. Differences were considered statistically significant at *p* ≤ 0.05.

## SUPPLEMENTARY MATERIALS FIGURES AND TABLES



## References

[1] Arndt CA, Stoner JA, Hawkins DS, Rodeberg DA, Hayes-Jordan AA, Paidas CN, Parham DM, Teot LA, Wharam MD, Breneman JC, Donaldson SS, Anderson JR, Meyer WH (2009). Vincristine, actinomycin, and cyclophosphamide compared with vincristine, actinomycin, and cyclophosphamide alternating with vincristine, topotecan, and cyclophosphamide for intermediate-risk rhabdomyosarcoma: children's oncology group study D9803. J Clin Oncol.

[2] Dantonello TM, Int-Veen C, Harms D, Leuschner I, Schmidt BF, Herbst M, Juergens H, Scheel-Walter HG, Bielack SS, Klingebiel T, Dickerhoff R, Kirsch S, Brecht I (2009). Cooperative trial CWS-91 for localized soft tissue sarcoma in children, adolescents, and young adults. J Clin Oncol.

[3] Oberlin O, Rey A, Sanchez de TJ, Martelli H, Jenney ME, Scopinaro M, Bergeron C, Merks JH, Bouvet N, Ellershaw C, Kelsey A, Spooner D, Stevens MC (2012). Randomized comparison of intensified six-drug versus standard three-drug chemotherapy for high-risk nonmetastatic rhabdomyosarcoma and other chemotherapy-sensitive childhood soft tissue sarcomas: long-term results from the International Society of Pediatric Oncology MMT95 study. J Clin Oncol.

[4] Sultan I, Ferrari A (2010). Selecting multimodal therapy for rhabdomyosarcoma. Expert Rev. Anticancer Ther.

[5] Malempati S, Hawkins DS (2012). Rhabdomyosarcoma: review of the Children's Oncology Group (COG) Soft-Tissue Sarcoma Committee experience and rationale for current COG studies. Pediatr. Blood Cancer.

[6] Casanova M, Ferrari A (2011). Pharmacotherapy for pediatric soft-tissue sarcomas. Expert Opin. Pharmacother.

[7] Chen X, Stewart E, Shelat AA, Qu C, Bahrami A, Hatley M, Wu G, Bradley C, McEvoy J, Pappo A, Spunt S, Valentine MB, Valentine V (2013). Targeting oxidative stress in embryonal rhabdomyosarcoma. Cancer Cell.

[8] Shern JF, Chen L, Chmielecki J, Wei JS, Patidar R, Rosenberg M, Ambrogio L, Auclair D, Wang J, Song YK, Tolman C, Hurd L, Liao H (2014). Comprehensive genomic analysis of rhabdomyosarcoma reveals a landscape of alterations affecting a common genetic axis in fusion-positive and fusion-negative tumors. Cancer Discov.

[9] Barr FG, Qualman SJ, Macris MH, Melnyk N, Lawlor ER, Strzelecki DM, Triche TJ, Bridge JA, Sorensen PH (2002). Genetic heterogeneity in the alveolar rhabdomyosarcoma subset without typical gene fusions. Cancer Res.

[10] Van Gaal JC, Flucke UE, Roeffen MH, de Bont ES, Sleijfer S, Mavinkurve-Groothuis AM, Suurmeijer AJ, Van Der Graaf WT, Versleijen-Jonkers YM (2012). Anaplastic lymphoma kinase aberrations in rhabdomyosarcoma: clinical and prognostic implications. J Clin Oncol.

[11] Wachtel M, Dettling M, Koscielniak E, Stegmaier S, Treuner J, Simon-Klingenstein K, Buhlmann P, Niggli FK, Schafer BW (2004). Gene expression signatures identify rhabdomyosarcoma subtypes and detect a novel t(2;2)(q35;p23) translocation fusing PAX3 to NCOA1. Cancer Res.

[12] Sumegi J, Streblow R, Frayer RW, Dal CP, Rosenberg A, Meloni-Ehrig A, Bridge JA (2010). Recurrent t(2;2) and t(2;8) translocations in rhabdomyosarcoma without the canonical PAX-FOXO1 fuse PAX3 to members of the nuclear receptor transcriptional coactivator family. Genes Chromosomes Cancer.

[13] Roskoski R (2013). Anaplastic lymphoma kinase (ALK): structure, oncogenic activation, and pharmacological inhibition. Pharmacol. Res.

[14] Kelleher FC, McDermott R (2010). The emerging pathogenic and therapeutic importance of the anaplastic lymphoma kinase gene. Eur. J Cancer.

[15] Mosse YP, Laudenslager M, Longo L, Cole KA, Wood A, Attiyeh EF, Laquaglia MJ, Sennett R, Lynch JE, Perri P, Laureys G, Speleman F, Kim C (2008). Identification of ALK as a major familial neuroblastoma predisposition gene. Nature.

[16] Pillay K, Govender D, Chetty R (2002). ALK protein expression in rhabdomyosarcomas. Histopathology.

[17] Bonvini P, Zin A, Alaggio R, Pawel B, Bisogno G, Rosolen A (2013). High ALK mRNA expression has a negative prognostic significance in rhabdomyosarcoma. Br. J Cancer.

[18] Mosse YP (2016). Anaplastic Lymphoma Kinase as a Cancer Target in Pediatric Malignancies. Clin Cancer Res.

[19] Cessna MH, Zhou H, Sanger WG, Perkins SL, Tripp S, Pickering D, Daines C, Coffin CM (2002). Expression of ALK1 and p80 in inflammatory myofibroblastic tumor and its mesenchymal mimics: a study of 135 cases. Mod. Pathol.

[20] Li XQ, Hisaoka M, Shi DR, Zhu XZ, Hashimoto H (2004). Expression of anaplastic lymphoma kinase in soft tissue tumors: an immunohistochemical and molecular study of 249 cases. Hum. Pathol.

[21] Corao DA, Biegel JA, Coffin CM, Barr FG, Wainwright LM, Ernst LM, Choi JK, Zhang PJ, Pawel BR (2009). ALK expression in rhabdomyosarcomas: correlation with histologic subtype and fusion status. Pediatr. Dev. Pathol..

[22] Yoshida A, Shibata T, Wakai S, Ushiku T, Tsuta K, Fukayama M, Makimoto A, Furuta K, Tsuda H (2013). Anaplastic lymphoma kinase status in rhabdomyosarcomas. Mod. Pathol.

[23] Soda M, Choi YL, Enomoto M, Takada S, Yamashita Y, Ishikawa S, Fujiwara S, Watanabe H, Kurashina K, Hatanaka H, Bando M, Ohno S, Ishikawa Y (2007). Identification of the transforming EML4-ALK fusion gene in non-small-cell lung cancer. Nature.

[24] Kwak EL, Bang YJ, Camidge DR, Shaw AT, Solomon B, Maki RG, Ou SH, Dezube BJ, Janne PA, Costa DB, Varella-Garcia M, Kim WH, Lynch TJ (2010). Anaplastic lymphoma kinase inhibition in non-small-cell lung cancer. N. Engl. J Med..

[25] Peron M, Lovisa F, Poli E, Basso G, Bonvini P (2015). Understanding the Interplay between Expression, Mutation and Activity of ALK Receptor in Rhabdomyosarcoma Cells for Clinical Application of Small-Molecule Inhibitors. PLoS One.

[26] Ladanyi M, Cavalchire G, Morris SW, Downing J, Filippa DA (1994). Reverse transcriptase polymerase chain reaction for the Ki-1 anaplastic large cell lymphoma-associated t(2;5) translocation in Hodgkin's disease. Am. J Pathol.

[27] Lamant L, Gascoyne RD, Duplantier MM, Armstrong F, Raghab A, Chhanabhai M, Rajcan-Separovic E, Raghab J, Delsol G, Espinos E (2003). Non-muscle myosin heavy chain (MYH9): a new partner fused to ALK in anaplastic large cell lymphoma. Genes Chromosomes Cancer.

[28] Lamant L, Dastugue N, Pulford K, Delsol G, Mariame B (1999). A new fusion gene TPM3-ALK in anaplastic large cell lymphoma created by a (1;2) (q25;p23) translocation. Blood.

[29] Touriol C, Greenland C, Lamant L, Pulford K, Bernard F, Rousset T, Mason DY, Delsol G (2000). Further demonstration of the diversity of chromosomal changes involving 2p23 in ALK-positive lymphoma: 2 cases expressing ALK kinase fused to CLTCL (clathrin chain polypeptide-like). Blood.

[30] Ma Z, Hill DA, Collins MH, Morris SW, Sumegi J, Zhou M, Zuppan C, Bridge JA (2003). Fusion of ALK to the Ran-binding protein 2 (RANBP2) gene in inflammatory myofibroblastic tumor. Genes Chromosomes Cancer.

[31] Kwak EL, Bang YJ, Camidge DR, Shaw AT, Solomon B, Maki RG, Ou SH, Dezube BJ, Janne PA, Costa DB, Varella-Garcia M, Kim WH, Lynch TJ (2010). Anaplastic lymphoma kinase inhibition in non-small-cell lung cancer. N Engl J Med.

[32] Katayama R, Khan TM, Benes C, Lifshits E, Ebi H, Rivera VM, Shakespeare WC, Iafrate AJ, Engelman JA, Shaw AT (2011). Therapeutic strategies to overcome crizotinib resistance in non-small cell lung cancers harboring the fusion oncogene EML4-ALK. Proc. Natl. Acad. Sci U. S. A..

