# Supplementary material for: Anaplastic lymphoma kinase aberrations correlate with metastatic features in pediatric rhabdomyosarcoma
Source: Oncotarget. 2016 Jul 1;7(37):58903–14. doi: 10.18632/oncotarget.10368 (PMC5312284; doi:10.18632/oncotarget.10368)
Supplement: Supplementary file 1 [file oncotarget-07-58903-s001.pdf]

Anaplastic lymphoma kinase aberrations correlate with metastatic features in pediatric rhabdomyosarcoma

Supplementary Materials

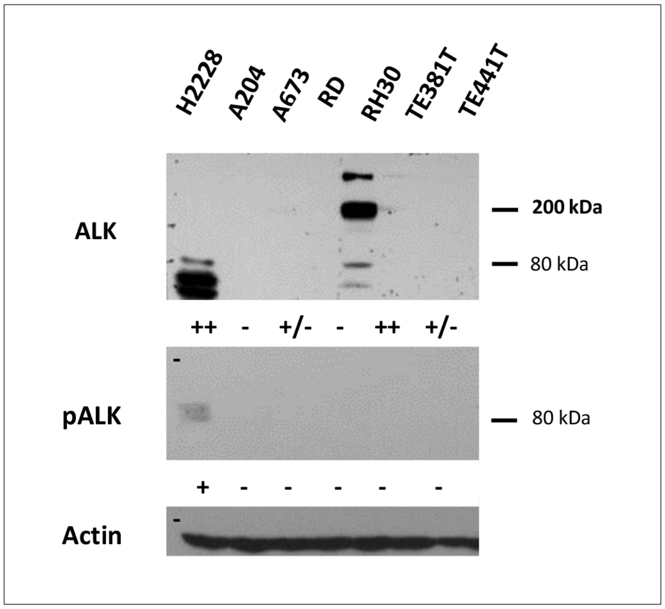

Supplementary Figure S1: ALK protein expression in RMS cell lines as detected by Western blotting. Cell line H2228 was utilized as a positive control for ALK rearrangement, whereas A204 was utilized as a negative control.

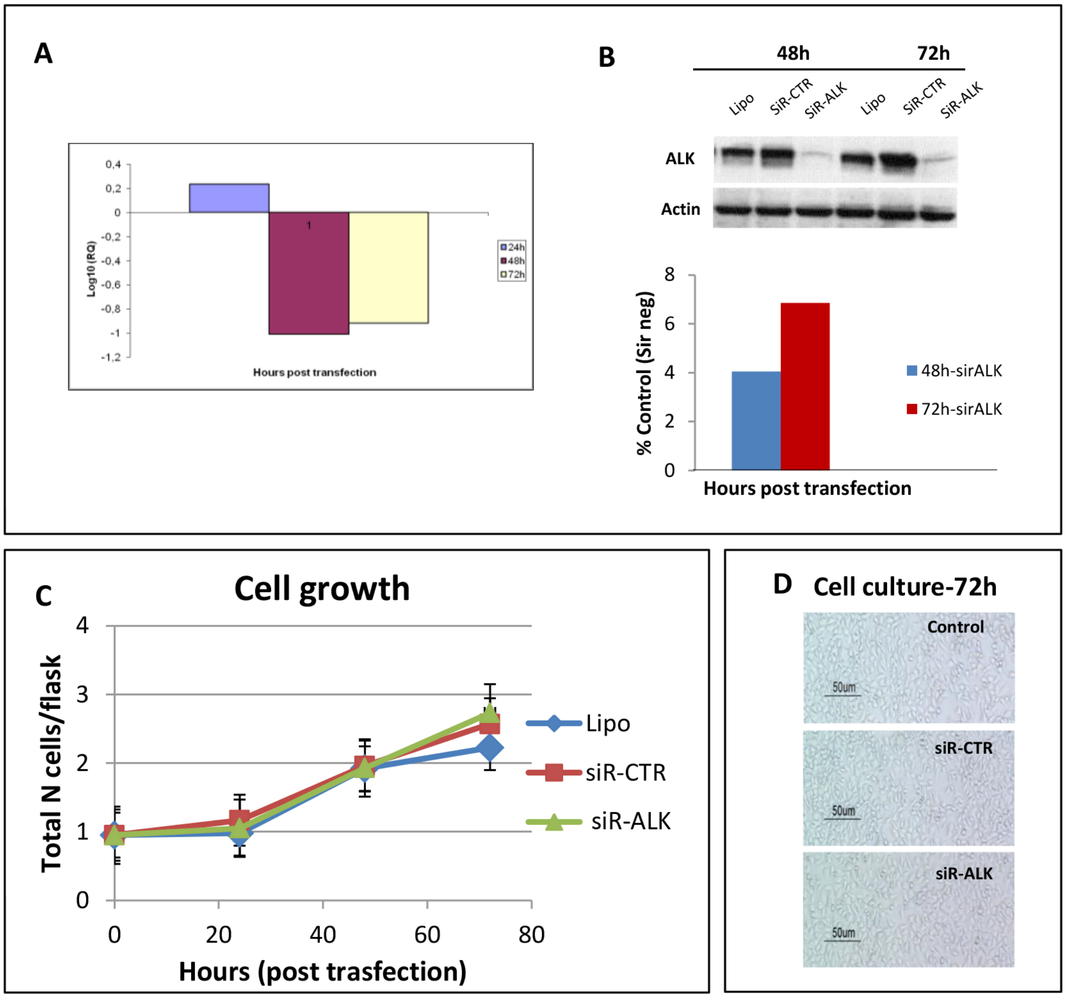

Supplementary Figure S2: siRNA-mediated down-modulation of ALK mRNA. (**A**) and protein (**B**) expression as detected by qRT-PCR and Western blotting, respectively. Lack of effects of ALK down-modulation on RH30 cell proliferation (**C**) and morphology (**D**).

Supplementary Table S1: Characterization of RMS cell lines for ALK protein expression

Cell line	Histotype	Fusion status	IHC ALK	ALK FISH	ALK WB	pALK WB
RH30	ARMS	PAX3-FOXO1	Positive	5–12 signals	Positive (200+120kDa)+80 kDa	Negative
RH4	ARMS	PAX3-FOXO1	Negative	4–6 signals	Negative (faint)	Negative
A204	Rhabdoid	negative	Negative	3 signals	Negative	Negative
RD	ERMS	negative	Negative	2 signals	Negative	Negative
TE381	ERMS	negative	Positive	6 signals	Positive (faint)	Negative
RH36	ERMS	negative	Negative	2–3 signals	Negative	Negative
RH18	ARMS	negative	Negative	2–3 signals	Negative	Negative
RD-M1 xenograft	ERMS	negative	Negative	n.v.	Negative	Negative
RD-M2 xenograft	ERMS	negative	Negative	n.v.	Negative	Negative
TE671	ERMS	negative	Negative	2 signals	Negative	Negative
CW9019	ARMS	PAX7-FOXO1	Negative	2 signals	Negative	Negative
TE441	RMS	negative	Negative	2 signals	Negative	Negative
